# Trends in Intake of Energy and Total Sugar from Sugar-Sweetened Beverages in the United States among Children and Adults, NHANES 2003–2016

**DOI:** 10.3390/nu11092004

**Published:** 2019-08-25

**Authors:** Bernadette P. Marriott, Kelly J. Hunt, Angela M. Malek, Jill C. Newman

**Affiliations:** 1 Department of Medicine, Medical University of South Carolina, 114 Doughty Street, Charleston, SC 29425, USA; 2 Department of Psychiatry and Behavioral Sciences, Medical University of South Carolina, Charleston, SC 29425, USA; 3 Department of Public Health Sciences, Medical University of South Carolina, 135 Cannon Street, Charleston, SC 29425, USA

**Keywords:** NHANES, SSBs, energy, total sugar, trends, children, adults

## Abstract

Consumption of sugar-sweetened beverages (SSBs) increases total caloric intake, is linked to cardiometabolic outcomes as well as dental caries, and sugar in SSBs is associated with mortality and frailty among adults. We describe energy and total sugar intake trends among the United States (US) population from SSBs, soft drinks, other beverage groups, and the total diet based on the first 24-h recall data from the National Health and Nutrition Examination Survey (NHANES) cycles (2003–2004 through 2015–2016). SSBs included soft drinks, sports drinks, energy drinks, and fruit drinks, but excluded sports beverages with protein and sweetened teas/coffees. Among the total population (age ≥2 years: 57,026), energy intake from SSBs declined significantly from 183.9 ± 6.9 mean kcal/d (±SE) in 2003–2004 to 95.0 ± 3.5 in 2015–2016, while total sugar intake declined from 43.6 ± 1.7 mean g/d to 22.3 ± 0.8 (*p*-trend < 0.0001). Decreases were found for energy and total sugar intake, as well as percentage of energy and total sugar intake from SSBs, soft drinks, and all beverages for all age groups examined (≥2, 2–19, ≥20 years) (*p*-trend < 0.0001). From 2003 to 2016, energy and sugar intake from all beverages, SSBs, soft drinks, and the total diet decreased among the total population, children, and adults.

## 1. Introduction

Sugar-sweetened beverages (SSBs) contribute approximately 39% of the added sugars consumed in the United States (US) [1] and account for the largest source of added sugars in most Western countries. Consumption of SSBs increases total caloric intake and has been linked to cardiometabolic outcomes, including metabolic syndrome, weight gain, and type 2 diabetes, as well as dental caries in the US and globally [2,3,4,5,6]. Consumption of SSBs has also been associated with preventable death/disability among adults [7]. US National Health and Nutrition Examination Survey (NHANES) data from 1999–2006 indicated that SSB intake during pregnancy was associated with significantly higher total energy intake and lower diet quality [8]. Recent analyses of SSB consumption by 37,716 men from the Health Professional’s Follow-up Study and 80,647 women from the Nurses’ Health Study, after adjustments for diet and lifestyle factors, found that SSB consumption was positively associated with cardiovascular disease (CVD) and all-cause mortality in a graded dose-response manner [9]. Using cross-sectional data from the National Health Interview Survey (NHIS), Park et al. reported statistically significant regional geographic differences in SSB intake among US adults, with the highest intake among adults living in the Northeast [10]. An 18-month clinical trial demonstrated that masked replacement of SSBs with sugar-free alternatives reduced both body weight and fat gain among children, thereby providing further demonstration of the link between intake of SSBs and concerns about childhood obesity [11].

The 2015–2020 Dietary Guidelines for Americans recommended that the US population increase intake of water, decrease consumption of beverages with added sugar, and limit added sugar intake to <10% of the day’s total calories [3]. A 2013 study by Kit et al. examined trends in consumption of SSBs by age group, sex, race/ethnicity, location (in and out of the home), eating occasion, and type and daily frequency of SSBs consumed using the 1999–2010 NHANES cycles [5]. Among both adults (≥20 years of age) and youth (2–19 years old), a significant decrease in percent of daily energy intake from SSBs was reported from 1999 to 2010 [5]. Similarly, daily energy intake from SSBs for both groups experienced a statistically significant decrease over time [5]. More recently, trends in beverage consumption were assessed by Bleich et al. by age group, sex, race/ethnicity, and type of SSB using NHANES cycles 2003–2004 through 2013–2014. This study found a significant decrease in energy intake from SSBs over time by age group for those 2–5, 6–11, 12–19, 20–39, and 40–59 years of age; however, no difference was found among older individuals (≥60 years of age) [12].

While trends in the per capita consumption of SSB calories has been reported for children and adults based on recent NHANES cycles [5,12], less is known regarding potential differences in trends of SSB consumption and intake of energy and total sugar by age groups. Adding to the complexity of understanding the role of SSBs in caloric and sugar intake is the diversity of definitions for SSBs [2,12,13], as well as debate over which sugars in the diet should be of concern (total sugar, free sugar, or added sugar), and thus should be a global focus for public health [14].

The purpose of the current study was to describe energy and total sugar consumption trends for SSBs, soft drinks, and total beverages, as well as the total diet in the US population among various age groups using NHANES 2003–2004 to 2015–2016 cycle data.

## 2. Materials and Methods

### 2.1. Study Population

The NHANES dietary intake data over one 24-h period (Day 1) across seven consecutive cycles [2003–2004 (C), 2005–2006 (D), 2007–2008 (E), 2009–2010 (F), 2011–2012 (G), 2013–2014 (H), 2015–2016 (I)] was used for this study [15]. NHANES is a nationally representative cross-sectional survey administered by the Centers for Disease Control and Prevention (CDC) National Center for Health Statistics (NCHS) to a sample of noninstitutionalized, civilian US residents to collect dietary data using a complex, stratified, multistage probability cluster sampling design [16].

After removal of data that did not meet the criteria for inclusion based on an NHANES variable for reliable dietary data (27 individuals), 63,048 participants had complete 24-h dietary intake data. A total of 6022 individuals were further excluded [pregnant or lactating women (*n* = 1282), and children <2 years old (*n* = 4740)]. The analytic sample thus included a total of 57,026 participants aged ≥2 years (*n* = 5534 children aged 2–5, *n* = 7378 children aged 6–11, *n* = 10,178 children aged 12–19, and *n* = 33,936 adults aged ≥20), of whom 28,747 were male participants (50.4%). See participant flow chart found in Online Supplemental Figure S1.

### 2.2. NHANES Dietary Interview

The current study is focused on the first 24-h dietary recall data collection that was conducted in-person. Specifically, this study used the food and beverage items reported consumed by participants between midnight and midnight 24 h prior to the NHANES dietary interview collected using a computer-assisted dietary interview software program, United States Department of Agriculture’s (USDA) automated multiple-pass method (AMPM). Consumption data were reported for children aged ≤5 years by proxy respondents, whereas for children aged 6–11 years of age, proxy-assisted interviews were conducted. We combined the data in the dietary interview component of the NHANES [What We Eat in America (WWEIA)] with the relevant items in the USDA’s Food and Nutrient Database for Dietary Studies (FNDDS). The FNDDS provides gram amounts to enable conversion of NHANES food and beverage intake information into estimated nutrient values [17]. We further grouped the reported intake of food and beverage items into specific beverage categories described below.

### 2.3. Sugar-Sweetened Beverages Definition and Categories

For this study, SSBs included soft drinks, sports drinks, energy drinks, and fruit drinks, but excluded sports beverages with protein, sweetened teas/coffees, and other items, such as low calorie beverages, 100% fruit juices etc., which were grouped separately similar to the US Dietary Guidelines for Americans Committee Report, 2015–2020 and the US Dietary Guidelines, 2015–2020. Soft drinks were defined as regular sweetened carbonated soda. The “all beverages” category included all beverage groups, such as SSBs, sports beverages with protein, and milk-based, dairy-based, protein-based, low/no calorie beverages, and alcoholic beverages, and therefore conformed to the definition of “beverage” by the USDA Food Surveys Research Group [18]. Total diet was defined as the entire diet, including foods, beverages, accompaniments, and “other”. Items added to food or beverages that contained calories, such as condiments, sauces, cream, etc., were classed as “accompaniments”; while “other” were items added to food or beverages that contained low or no calories, such as a packet of low calorie sweetener. Classification of reported beverages as beverage types and SSBs was performed by one author (B.P.M.).

### 2.4. Statistical Analysis

The average percentage of energy intake and total sugar intake over time in the US was reported for the entire population ≥2 years and for the age groups 6–11, 12–19, 2–19, and ≥20 years for both sexes combined. The percent of SSB consumption (% of energy intake from total diet coming from SSBs) was categorized in tiers as: 0 ounces (non-SSB consumers), >0–12 ounces, >12–24 ounces, and >24 ounces.

All analyses accounted for the clustered sampling design and oversampling, with adjustment across the seven continuous NHANES cycles for differential non-coverage and non-response [19,20,21,22]. For total energy and total sugar consumption, means and standard errors (SE) were reported for average nutrient intake; mean percentage ± SE of total diet was also reported. SAS complex survey-specific procedures were used for all analyses to account for NHANES survey design and suggested analytic methodology. Trends over time (cycles) were examined using survey linear regression and survey logistic regression that included age subgroup analyses for total energy and total sugar consumption. All regression analyses controlled for multiple cycles to measure trends. Estimates for adults (aged ≥20 years) were age-adjusted by the direct method to the year 2010 US Census Population. Since some residual distributions from the regression analyses were not normally distributed, outcome variables were transformed in several different ways to change the measurement scale of the distribution. Transformations performed included natural log, log + 1, square root, and cube root transformations. Regression analyses were run on the transformed outcomes as a sensitivity analysis.

The resulting trend of highly significant differences in means over the cycles was consistent in every modeling scenario. Line graphs were created to show temporal trends for mean energy intake, mean total sugar intake, and percent of SSB consumption by tiers of consumption for select age groups. Mean ± standard error (SE) is shown for all values.

SAS version 9.4 was used to conduct all analyses (SAS Institute; Cary, USA) and a *p*-value ≤ 0.05 was considered statistically significant. The Medical University of South Carolina did not require Institutional Review Board approval as the study constituted secondary data analysis and the study was not considered human subjects research.

## 3. Results

### 3.1. Trends in Estimated Mean Amount of Energy Intake by Age Group

**Figure 1:**
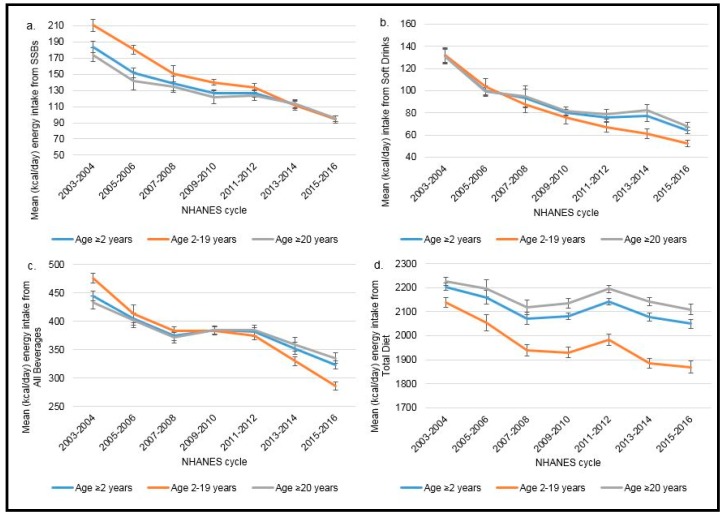
Trends in energy intake from (**a**) sugar-sweetened beverages (SSBs); (**b**) soft drinks; (**c**) all beverages; and (**d**) total diet in the US by age group.

Trends in energy intake from SSB consumption, soft drink consumption, total beverage consumption, and the total diet over the 14-year period (2003–2016) are presented in Figure 1a–d for the total population (aged ≥2 years), children aged 2–19, and adults aged ≥20 years. [Supplemental Table S1 includes mean energy intake values that correspond to Figure 1 with data for two additional age categories (children aged 6–11, children aged 12–19).] Estimated energy intake from SSBs, soft drinks, all beverages, and the total diet declined among all age groups studied (≥2, 2–19, 6–11, 12–19, ≥20) from NHANES cycle 2003–2004 through NHANES cycle 2015–2016. In this time period, energy intake from SSBs decreased by more than half among children (2–19 years), and by almost half among adults (≥20 years). Energy intake from soft drinks alone declined 59% to 61% among children depending on age category (2–19, 6–11, 12–19 years) and 48% among adults (≥20 years). Energy intake from all beverages also declined substantially among children, from 36% to 42% depending on age category, and to a lesser extent among adults (23%). Declines in energy intake in the total diet were more moderate at 13% among children aged 2–19 and 5% among adults aged ≥20 years.

NHANES dietary data over 7 cycles (2003–2016) was used (*n* = 57,026; 23,090 children aged 2–19, *n* = 33,936 adults aged ≥20 years). All tests for significance for a linear trend were statistically significant at *p* < 0.0001 based on linear regression for survey data. Estimates for adults aged ≥20 years were age-adjusted by the direct method to the year 2010 US Census Population. Mean ± SE (kcal/day) energy intake is presented by NHANES cycle and age group from: (a) SSBs (sugar-sweetened beverages), defined as soft drinks, sports drinks, energy drinks, and fruit drinks, excluding sports beverages with protein, sweetened teas/coffees, and other items; (b) soft drinks, defined as regular sweetened carbonated soda; (c) all beverages; and (d) the total diet.

### 3.2. Trends in Average Percentage of Estimated Daily Energy Intake by Age Group

Trends in average percentage of daily energy intake from SSB consumption, soft drink consumption, and total beverage consumption over the 14-year period (2003–2016) are presented in Table 1 for the total population (aged ≥2 years), children aged 2–19, children aged 6–11, children aged 12–19, and adults aged ≥20 years. In comparison to estimates of absolute levels provided in Figure 1a–d, these estimates report the percentage (or proportion) of total energy coming from each category examined while accounting for total daily energy intake. Average percentage of estimated daily energy intake from SSBs, soft drinks, and all beverages declined among all age groups across NHANES cycles 2003–2016. Average percentage of energy intake from SSBs decreased among children and among adults, with changes representing between a 40% and 50% decline in average percentage of energy intake from SSBs from 2003 to 2016. Changes in the average percentage of energy intake from soft drinks declined 56% among children aged 2–19 years and 44% among adults aged ≥20 years, while changes in the average percentage of energy intake from all beverages declined 30% among children aged 2–19 years and 17% among adults aged ≥20 years.

### 3.3. Trends in Estimated Mean Total Sugar Intake by Age Group

**Figure 2:**
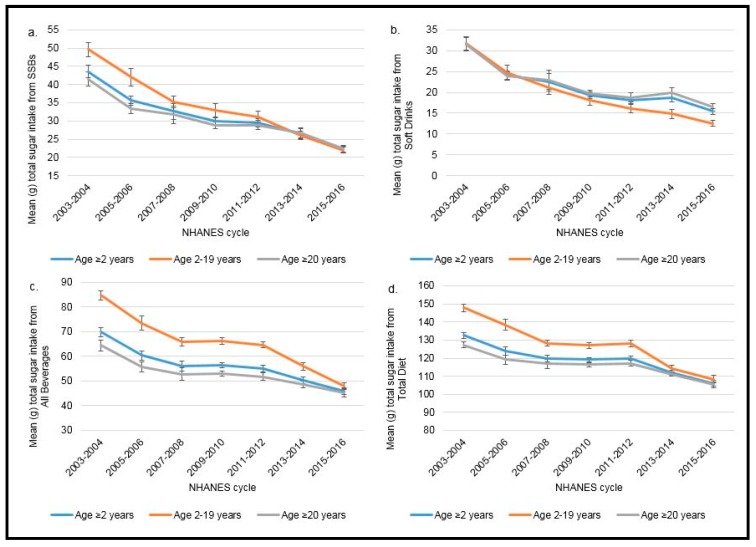
Trends in total sugar intake (g) from (**a**) SSBs; (**b**) soft drinks; (**c**) all beverages; and (**d**) the total diet in the US by age group.

Trends in total sugar intake from SSBs, soft drinks, all beverages, and the total diet from 2003–2016 are presented in Figure 2a–d for the total population (aged ≥2 years), children aged 2–19, and adults aged ≥20 years. (Supplemental Table S2 includes the numerical total sugar intake values.) Estimated total sugar intake from SSBs, soft drinks, all beverages, and the total diet declined among all age groups across the 14-year time frame. From 2003–2016, total sugar intake from SSBs decreased by 51% to 57% among children depending on age category (2–19, 6–11, 12–19 years), and by 46% among adults (≥20 years). Total sugar intake from soft drinks alone declined 61% among children aged 2–19 years and 48% among adults (≥20 years). Total sugar intake from all beverages also declined substantially, 43% among children and 30% among adults, with declines in total sugar intake in the total diet also being substantial (27% among children and 17% among adults).

NHANES dietary data over 7 cycles (2003–2016) was used (*n* = 57,026; 23,090 children aged 2–19, *n* = 33,936 adults aged ≥20 years). All tests for significance for a linear trend were statistically significant at *p* < 0.001 based on linear regression for survey data. Estimates for adults aged ≥20 years were age-adjusted by the direct method to the 2010 US Census Population. Mean percentage ± SE (g) total sugar intake is presented by NHANES cycle and age group from: (a) SSBs (sugar-sweetened beverages), defined as soft drinks, sports drinks, energy drinks, and fruit drinks, excluding sports beverages with protein, sweetened teas/coffees, and other items; (b) soft drinks, defined as regular sweetened carbonated soda; (c) all beverages; and (d) the total diet.

### 3.4. Trends in Average Percentage of Total Sugar Intake from Consumption of SSBs, Soft Drinks, and All Beverages by Age Group

Trends in average percentage of total sugar intake from SSB consumption, soft drink consumption, and total beverage consumption over the 14-year period (2003–2016) are presented in Table 2. Average percentage of total sugar intake from SSBs, soft drinks, and all beverages declined among all age groups over this time period. Average percentage of total sugar intake from SSBs decreased 40% among children aged 2–19 years and 38% among adults from 2003 to 2016. Changes in the average percentage of total sugar intake from soft drinks alone declined 47% among children aged 2–19 years and 41% among adults, while changes in the average percentage of total sugar intake from all beverages declined 23% among children aged 2–19 years and 19% among adults aged ≥20 years. 

### 3.5. Percentage of SSB Consumers and Non-Consumers Over Time by Age Group and Tier of Consumption

**Figure 3:**
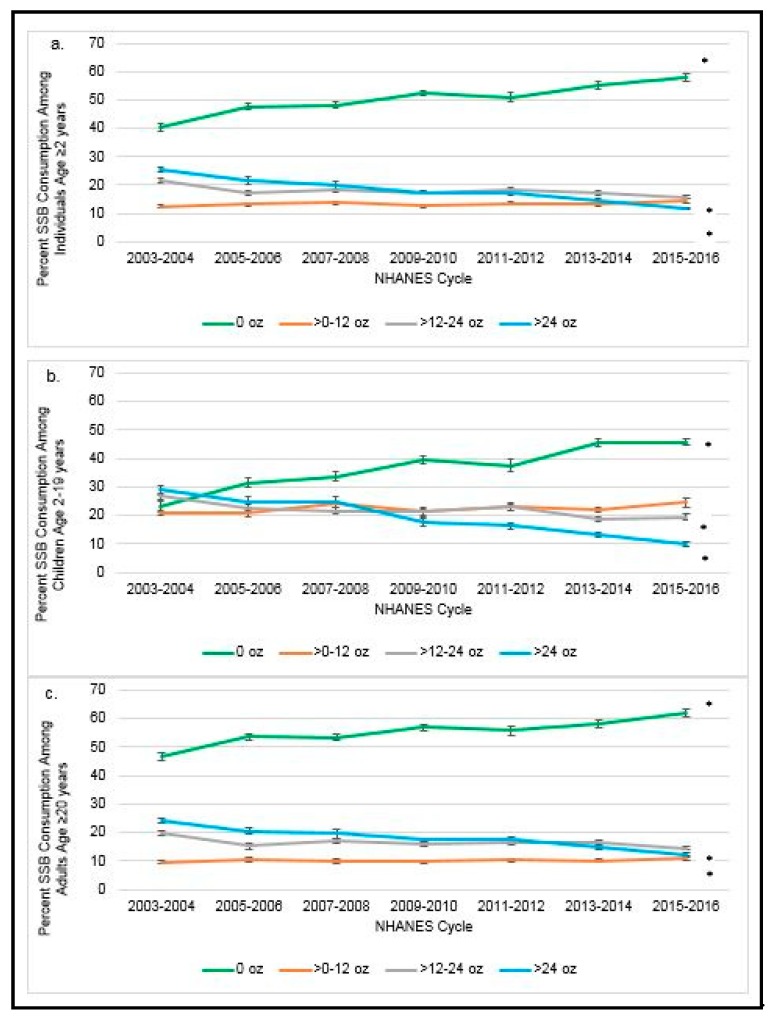
Percentage of the US population by age group (**a**) ≥2 yr; (**b**) 2–19 yr; and (**c**) ≥20 yr and tiers of SSB consumption over time.

The percentage of SSB consumers and non-consumers over time by tier of SSB consumption is presented in Figure 3a–c by age group. (Supplemental Table S3 includes corresponding numeric values.) Among the entire sample aged ≥2 years (see Figure 3a), the percentage of non-SSB consumers appears to have increased from 2003–2016 from 40.3 ± 1.3% to 58.2 ± 1.3%, whereas the percentage who reported consuming >12–24 oz. and ≥24 oz. of SSBs per day decreased from 21.6 ± 0.7% to 15.8 ± 0.1% and 25.6 ± 1.0% to 11.7 ± 0.5%, respectively (all *p*-trend < 0.0001). In contrast, the percentage of ≥2 year olds consuming >0–12 oz. of SSBs appears to have remained steady from 2003 through 2016. Similarly, among children aged 2–19 years, the percentage of non-SSB consumers has increased from 23.1 ± 1.7% in 2003–2004 to 45.8 ± 1.2% in 2015–2016, and the percentage consuming >12–24 oz. and ≥24 oz. of SSBs has declined (see Figure 3b). Among adults aged ≥20 years, the percentage who do not consume SSBs also increased from 45.6 ± 1.3% in 2003–2004 to 62.2 ± 1.4% in 2015–2016 (*p*-trend < 0.0001), while the percentage consuming >12–24 oz. (*p*-trend = 0.0002) and ≥24 oz. (*p*-trend < 0.0001) of SSBs has declined (see Figure 3c). Again, among children aged 2–19 years (Figure 3b) and adults aged ≥20 years (Figure 3c) the percentage consuming >0–12 oz. of SSBs has remained relatively steady, with the highest percentages among those 2–19 years.

NHANES dietary data over 7 cycles (2003–2016) was used (*n* = 57,026; 23,090 children aged 2–19, *n* = 33,936 adults aged ≥20). Tests for significance for a linear trend were statistically significant at *p* < 0.001 based on linear regression for survey data as indicated by an asterisk (*). Estimates for adults aged ≥20 years were age-adjusted by the direct method to the year 2010 US Census Population. Percentage ± SE SSB (sugar-sweetened beverages) consumption is displayed by NHANES cycle among: (a) Individuals age ≥2 years, (b) children age 2–19 years, and (c) adults age ≥20 years. SSBs (sugar-sweetened beverages) were defined as soft drinks, sports drinks, energy drinks, and fruit drinks, excluding sports beverages with protein, sweetened teas/coffees, and other items.

## 4. Discussion

Energy and sugar intake from all beverages, SSBs, soft drinks, and the total diet decreased for children and adults in the US from 2003 to 2016. Kit et al. [5] and, more recently, Bleich et al. [12] reported downward trends in SSB intake among both adults and children. We extend their findings to the 2015–2016 NHANES cycle and report a continued downward trend in the percentage of adults and children consuming SSBs. We also report a decline in energy intake, as well as average percentage of total diet energy intake from SSBs in adults and children, from NHANES 2003 through 2016. Of particular note is the increase in the proportion of individuals over the 14-year time span among all age groups who report that they are not consuming any SSBs. The percentage of non-consumers of SSBs almost doubled among children and increased by more than a third among adults over the time period studied.

We examined the percent of energy consumed from SSBs, soft drinks, and all beverages to provide context for SSB consumption as one part of caloric intake in the total diet. Because SSBs account for a large proportion of total sugar intake, we also examined trends in total sugar intake from SSBs, soft drinks, all beverages, and from the total diet, as well as the proportion of total sugars in the diet that came from SSBs, soft drinks, and all beverages. Average percentage of total sugar intake from SSBs, soft drinks, and all beverages declined among all age groups over this time period. These data underscore the impact these dietary beverage intake changes are having on total sugar consumption. The 2015–2020 Dietary Guidelines for Americans recommends a decrease in consumption of dietary items containing added sugar as an important component to improving the prospective health outcomes of the US population [3]. These data indicate that the current declining trends in beverage and total sugar intake, if they continue, are encouraging.

Recent analyses by the Cochrane group underscored the association between intake and portion size and estimated that reducing access to larger portions could reduce average daily energy intake by 22–29% among US adults [23]. We found an apparent decrease in beverage-based energy intake from 2003–2016. While this energy intake decrease is in part due to a significant increase in the percentage of non-consumers of SSBs across all age groups during the 14-year period, by addressing the tiers of consumption of beverages, we found that a significant reduction in intake of larger portion sizes among all age groups appears to have accounted in part for this decrease in beverage-based energy intake. However, reported intake of beverage sizes under 12 oz appears to have remained relatively constant within each age group over the same time period. These results are surprising because research has indicated, for example, that fountain drink portion sizes among three of the major fast-food chains have stayed relatively constant from 1998 through 2006, with a 12 oz. beverage (child size) being the smallest size available, with a large beverage containing 32 oz [24]. However, recent research has shown that local marketing campaigns to reduce SSB intake have been successful overall in significantly reducing the sales of SSBs, in terms of overall fluid ounces of product sold per week over a three-year period [25].

Based on the NHANES data from 2003 to 2016, we found that estimated total sugar intake from SSBs, soft drinks, all beverages, and the total diet decreased significantly among children and adults. The decrease in total sugar intake of over 50% among children was particularly encouraging, since clinical trial data from the Netherlands [11] and recent cross-sectional school-based survey data from Australia [26] demonstrated associations between sugar-containing beverage intake and obesity and oral health impacts among children and adolescents. Total sugar intake from all beverages declined 30% among adults with a 48% decrease in total sugar from soft drinks. Adult total sugar intake from the total diet also declined 17% across this time period. These data are promising, particularly in light of recent data from two adult prospective cohort studies (Health Professionals Follow-Up study and Nurses’ Health study) showing a positive association between long-term SSB consumption and mortality, primarily through CVD [9], and systematic reviews that link SSB consumption and weight gain among adults (cf., [27]).

Our study has a number of strengths; NHANES and FNDDS are nationally-representative databases that provide the most comprehensive dietary intake studies in the US, and our study reports on trends over a 14-year period. Our study has a number of limitations; underreporting of portion size of food and drink items has been cited as an issue for NHANES 24-h recall data, which may affect nutrient levels, and thus the overall intake by US children and adults may be underestimated [28]. However, the misreporting of food and beverage items has been reduced through use of the USDA’s AMPM for dietary intake data [18,29]. Additionally, by using data from only a single 24-h dietary recall, we did not consider within-person variability; hence, results should be viewed as representing intake on a single day, rather than a person’s usual intake. Finally, due to the cross-sectional design of NHANES, it is not possible to evaluate potential temporal relationships between factors.

## 5. Conclusions

Park et al. [10] have demonstrated that geographically, the frequency and type of SSB consumption significantly differs among US adults, such that public health messaging about SSB intake could be specifically targeted to have the largest impact. While the 2015 US Dietary Guidelines Advisory Committee focused on a reduction in added sugars in the US diet, globally, questions remain whether total, added, or free sugars should be the focus of public health messaging [14], with international policies focused on “free sugars” [30]. Approaches to reducing portion sizes [23], and successful community campaigns that have resulted in improved beverage choices at point of purchase [25], demonstrate that there are proven methods available to continue to reduce beverage energy and total sugar intake. With the long-term health outcomes continuing to emerge related to sugars and SSBs, going forward, positive educational messaging that incorporates understanding of what motivates SSB selection [31], and that underscores reduction of large daily portions and choice of beverage type, may enhance continued success in dietary caloric and sugar reduction among the US population.

## Figures and Tables

**Table 1:** Average percentage of energy intake over time in the US by age groups ^1–3^.

	2003–2004		2005–2006		2007–2008		2009–2010		2011–2012		2013–2014		2015–2016		*p*-Value ^4^
	%	SE	%	SE	%	SE	%	SE	%	SE	%	SE	%	SE	
**% Energy Intake from SSBs (kcal/day) ^5^**															
≥2 years old	8.1	0.3	6.8	0.2	6.5	0.4	5.8	0.2	5.8	0.2	5.3	0.2	4.5	0.1	<0.0001
2–19 years old	9.8	0.5	8.5	0.4	7.6	0.4	6.9	0.4	6.6	0.2	5.8	0.4	5.0	0.2	<0.0001
6–11 years old	8.6	0.6	6.7	0.4	6.9	0.4	5.9	0.3	6.0	0.3	5.1	0.4	4.9	0.3	<0.0001
12–19 years old	12.2	0.6	11.2	0.5	9.5	0.6	8.9	0.7	8.2	0.4	7.4	0.5	6.0	0.3	<0.0001
≥20 years old	7.5	0.3	6.2	0.2	6.1	0.4	5.4	0.2	5.5	0.2	5.1	0.2	4.3	0.2	<0.0001
**% Energy Intake from Soft Drinks (kcal/ day) ^5^**															
≥2 years old	5.7	0.3	4.4	0.2	4.3	0.4	3.6	0.2	3.5	0.2	3.5	0.2	3.0	0.1	<0.0001
2–19 years old	6.1	0.4	4.6	0.3	4.3	0.4	3.6	0.3	3.4	0.2	3.0	0.2	2.7	0.1	<0.0001
6–11 years old	5.3	0.5	3.1	0.2	3.6	0.3	2.7	0.2	2.7	0.3	2.4	0.3	2.4	0.2	<0.0001
12–19 years old	8.6	0.6	7.2	0.4	6.2	0.6	5.6	0.6	4.8	0.3	4.5	0.3	3.9	0.2	<0.0001
≥20 years old	5.5	0.3	4.3	0.2	4.3	0.4	3.6	0.2	3.6	0.2	3.7	0.2	3.1	0.1	<0.0001
**% Energy Intake from All Beverages (kcal/ day)**															
≥2 years old	19.6	0.4	18.3	0.4	17.8	0.3	18.0	0.3	17.6	0.3	16.6	0.4	15.4	0.3	<0.0001
2–19 years old	22.6	0.4	20.3	0.5	20.4	0.4	20.1	0.3	19.6	0.4	18.0	0.5	15.8	0.4	<0.0001
6–11 years old	21.1	0.7	18.1	0.7	19.0	0.4	18.8	0.4	18.1	0.5	16.8	0.4	15.2	0.5	<0.0001
12–19 years old	22.1	0.5	19.9	0.5	19.5	0.6	19.3	0.5	19.0	0.5	18.0	0.7	15.1	0.6	<0.0001
≥20 years old	18.5	0.4	17.5	0.5	16.9	0.4	17.3	0.3	17.0	0.3	16.1	0.4	15.3	0.4	<0.0001

^1^ Mean percentage ± standard error (SE). ^2^ Data source: NHANES dietary data over 7 cycles (2003–2016) was used (*n* = 57,026; *n* = 5534 children aged 2–5, *n* = 7378, children aged 6–11, *n* = 10,178 children aged 12–18, *n* = 33,936 adults aged ≥20 years). ^3^ Estimates for adults aged ≥20 years were age-adjusted by the direct method to the year 2010 US Census Population. ^4^ All tests for significance for a linear trend were obtained using linear regression for survey procedures. ^5^ SSBs (sugar-sweetened beverages) were defined as soft drinks, sports drinks, energy drinks, and fruit drinks, excluding sports beverages with protein, sweetened teas/coffees, and other items. Soft drinks were defined as regular sweetened carbonated soda. US, United States; SE, standard error; SSBs, sugar-sweetened beverages.

**Table 2:** Average Percentage of Total Sugar Intake over Time in the US by Age Groups ^1–3^.

	2003–2004		2005–2006		2007–2008		2009–2010		2011–2012		2013–2014		2015–2016		*p*-Value ^4^
	%	SE	%	SE	%	SE	%	SE	%	SE	%	SE	%	SE	
**% Total Sugar Intake from SSBs ^5^**															
≥2 years old	29.9	0.9	23.0	0.8	21.8	1.0	19.9	0.5	20.2	0.7	18.6	0.8	16.5	0.6	<0.0001
2–19 years old	30.7	1.2	27.2	1.1	24.7	1.1	22.4	1.0	22.4	1.0	19.9	1.0	18.5	0.6	<0.0001
6–11 years old	27.3	1.4	21.4	1.1	22.6	1.1	19.7	1.2	20.3	1.1	17.8	1.2	18.1	1.1	<0.0001
12–19 years old	38.7	1.8	36.7	1.5	30.9	1.6	29.0	1.6	28.4	1.5	25.2	1.2	22.9	0.9	<0.0001
≥20 years old	25.6	0.9	21.6	0.9	20.8	1.0	19.1	0.6	19.4	0.8	18.2	0.9	15.8	0.6	<0.0001
**% Total Sugar Intake from Soft Drinks ^5^**															
≥2 years old	19.5	0.8	15.4	0.6	14.8	1.1	12.7	0.5	12.7	0.6	12.7	0.6	11.1	0.4	<0.0001
2–19 years old	19.7	1.0	15.9	0.9	14.2	1.1	11.9	0.7	11.8	0.8	10.8	0.7	10.4	0.5	<0.0001
6–11 years old	17.4	1.3	10.6	0.8	12.1	1.0	9.3	0.8	9.5	1.0	8.3	0.9	9.1	0.8	<0.0001
12–19 years old	27.8	1.7	24.8	1.2	20.3	1.8	18.0	1.2	17.3	1.3	16.0	1.0	14.8	0.8	<0.0001
≥20 years old	19.4	0.8	15.2	0.6	15.0	1.1	12.9	0.5	13.0	0.7	13.3	0.7	11.4	0.5	<0.0001
**% Total Sugar Intake from All Beverages**															
≥2 years old	46.4	0.7	42.6	1.0	41.5	0.8	41.4	0.5	40.6	0.8	38.7	0.8	36.9	0.8	<0.0001
2–19 years old	55.4	0.7	50.5	1.0	49.8	1.0	49.2	0.6	48.9	0.8	46.1	1.0	42.8	1.2	<0.0001
6–11 years old	51.3	1.1	44.2	1.7	46.7	1.3	46.3	1.1	45.5	1.0	43.1	1.0	41.1	1.6	<0.0001
12–19 years old	58.9	1.1	55.0	0.9	52.0	1.2	51.5	0.8	52.1	1.0	48.9	1.2	44.7	1.5	<0.0001
≥20 years old	43.1	0.9	39.8	1.1	39.0	0.8	38.7	0.9	37.8	0.9	36.4	0.8	35.0	0.8	<0.0001

^1^ Mean percentage ± standard error (SE). ^2^ Data source: NHANES dietary data over 7 cycles (2003–2016) was used (*n* = 57,026; *n* = 5534 children aged 2–5, *n* = 7378, children aged 6–11, *n* = 10,178 children aged 12–18, *n* = 33,936 adults aged ≥20). ^3^ Estimates for adults aged ≥20 years were age-adjusted by the direct method to the year 2010 US Census Population. ^4^ All tests for significance for a linear trend were obtained using linear regression for survey procedures. ^5^ SSBs (sugar-sweetened beverages) were defined as soft drinks, sports drinks, energy drinks, and fruit drinks, excluding sports beverages with protein, sweetened teas/coffees, and other items. Soft drinks were defined as regular sweetened carbonated soda. US, United States; SE, standard error; SSBs, sugar-sweetened beverages.

## Supplementary Materials

The following are available online at https://www.mdpi.com/2072-6643/11/9/2004/s1, Figure S1: Flow chart defining study population and exclusions; Table S1: Trends in energy intake over time in the US by age groups; Table S2: Trends in total sugar intake over time in the US by age groups; Table S3: Percentage SSB consumers/non-consumers of total US population over time by age groups and tiers of SSB consumption.

## Abbreviations

AMPM	Automated Multiple-Pass Method
CDC	Center for Disease Control and Prevention
CVD	Cardiovascular disease
FNDDS	Food and Nutrient Database for Dietary Studies
NCHS	National Center for Health Statistics
NHANES	National Health and Nutrition Examination Survey
NHIS	National Healthy Interview Study
SE	Standard error
SSBs	Sugar-sweetened beverages
US	United States
USDA	United States Department of Agriculture
WWEIA	What We Eat in America
