# Supplementary material for: Trends in Intake of Energy and Total Sugar from Sugar-Sweetened Beverages in the United States among Children and Adults, NHANES 2003–2016
Source: Nutrients. 2019 Aug 25;11(9):2004. doi: 10.3390/nu11092004 (PMC6770750; doi:10.3390/nu11092004)
Supplement: Supplementary file 1 [file nutrients-11-02004-s001.pdf]

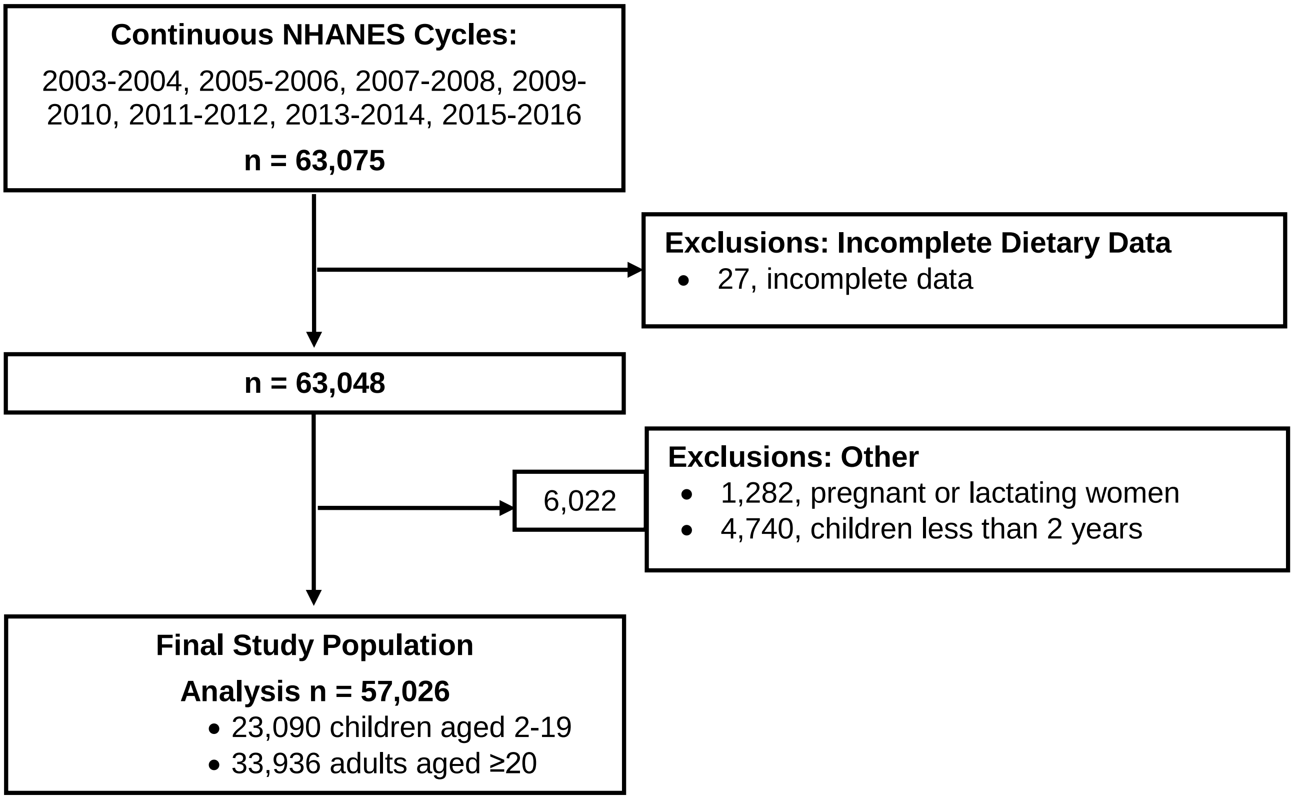

Figure S1: Flow chart defining study population and exclusions.

Table S1. Trends in Energy Intake over Time in the US by Age Groups [1-3]

	2003-2004		2005-2006		2007-2008		2009-2010		2011-2012		2013-2014		2015-2016		p-value [4]
	kcal/d	SE	kcal/d	SE	kcal/d	SE	kcal/d	SE	kcal/d	SE	kcal/d	SE	kcal/d	SE	<.0001
**Energy intake from SSBs [5]**															
≥2 years old	183.9	6.9	151.7	5.6	138.7	8.8	126.5	3.9	126.2	4.9	112.9	5.2	95.0	3.5	<.0001
2-19 years old	210.7	8.4	180.5	10.7	151.0	6.5	140.0	7.7	133.3	6.3	111.2	5.1	94.2	3.3	<.0001
6-11 years old	181.5	12.3	131.2	7.9	138.6	7.6	108.4	5.9	115.3	5.5	96.6	6.6	91.1	5.1	<.0001
12-19 years old	277.8	10.8	261.8	18.5	197.3	10.7	199.1	15.0	177.6	10.4	148.0	6.6	120.5	6.1	<.0001
≥20 years old	174.3	7.8	141.6	5.7	134.5	10.1	121.8	3.8	123.8	5.2	113.4	5.9	95.2	4.6	<.0001
**Energy intake from Soft Drinks [5]**															
≥2 years old	131.1	6.1	100.1	4.1	93.1	8.5	80.1	3.1	75.7	3.7	77.3	4.6	64.3	2.8	<.0001
2-19 years old	131.8	6.6	103.3	7.6	87.3	6.8	75.7	5.5	66.9	4.4	61.4	4.4	52.7	3.0	<.0001
6-11 years old	113.1	11.3	61.7	4.9	73.3	6.6	50.5	4.0	51.6	5.0	45.4	4.8	44.3	3.9	<.0001
12-19 years old	192.9	10.3	170.0	13.8	128.0	11.9	124.0	10.4	101.3	7.7	94.6	8.1	78.4	5.1	<.0001
≥20 years old	130.8	7.0	99.0	4.0	95.1	9.6	81.6	3.4	78.7	4.4	82.5	5.1	68.1	3.7	<.0001
**Energy intake from All Beverages**															
≥2 years old	444.8	9.0	404.2	10.9	374.1	7.9	384.0	6.3	381.5	8.0	351.9	10.7	322.9	6.9	<.0001
2-19 years old	475.9	8.5	413.2	15.1	382.8	7.1	383.1	6.7	374.4	7.5	329.8	7.5	286.1	6.7	<.0001
6-11 years old	443.0	16.5	354.8	16.6	359.7	9.7	346.2	7.8	343.9	10.8	314.0	7.2	282.1	10.1	<.0001
12-19 years old	516.5	10.4	471.7	22.1	404.8	11.2	421.8	11.4	407.0	12.3	358.9	11.6	299.6	12.9	<.0001
≥20 years old	433.7	11.4	401.1	11.8	371.0	9.9	384.3	7.4	384.0	9.5	359.1	12.3	334.8	9.4	<.0001
**Energy intake from Total Diet**															
≥2 years old	2203.9	15.5	2159.9	29.6	2072.5	24.8	2082.1	13.7	2141.3	12.6	2078.4	16.1	2050.4	18.7	<.0001
2-19 years old	2138.0	19.9	2053.5	34.2	1937.8	23.2	1929.4	21.9	1983.6	22.8	1884.9	21.1	1868.8	25.1	<.0001
6-11 years old	2121.4	49.7	1985.1	35.6	1928.0	27.9	1869.0	20.4	1986.8	30.7	1920.6	35.4	1907.4	28.2	.0001
12-19 years old	2350.1	41.2	2338.7	46.7	2145.0	32.9	2167.3	43.6	2175.0	44.9	2037.9	31.1	2032.0	51.7	<.0001
≥20 years old	2227.5	17.0	2197.5	34.0	2119.4	30.0	2134.5	20.7	2194.9	15.8	2141.2	17.3	2109.1	21.3	<.0001

[1] Mean ± standard error (SE) is shown for all values. [2] Data source: NHANES dietary data over 7 cycles (2003-2016) was used (n=57,026; n=5534 children aged 2-5, n=7378 children aged 6-11, n=10,178 children aged 12-18, n=33,936 adults aged ≥20). [3] Estimates for adults aged ≥20 years were age-adjusted by the direct method to the year 2010 US Census Population. [4] All tests for significance for a linear trend were obtained using linear regression for survey data. [5] SSBs (sugar-sweetened beverages) were defined as soft drinks, sports drinks, energy drinks, and fruit drinks, excluding sports beverages with protein, sweetened teas/coffees, and other items. Soft drinks were defined as regular sweetened carbonated soda. US, United States; SE standard error; SSBs, sugar-sweetened beverages.

Table S2. Trends in Total Sugar Intake over Time in the US by Age Groups [1-3]

	2003-2004		2005-2006		2007-2008		2009-2010		2011-2012		2013-2014		2015-2016		p-value[4]
	**g**	**SE**	**g**	**SE**	**g**	**SE**	**g**	**SE**	**g**	**SE**	**g**	**SE**	**g**	**SE**	<.0001
**Total Sugar intake from SSBs [5]**															
≥2 years old	43.6	1.7	35.6	1.3	32.6	2.1	29.9	0.9	29.5	1.1	26.5	1.3	22.3	0.8	<.0001
2-19 years old	49.6	1.9	42.0	2.5	35.3	1.6	32.9	1.8	31.2	1.5	26.1	1.2	22.0	0.8	<.0001
6-11 years old	42.6	3.0	30.4	1.8	32.3	1.9	25.4	1.4	27.0	1.3	22.7	1.6	21.0	1.2	<.0001
12-19 years old	65.7	2.5	61.2	4.4	46.3	2.7	46.9	3.6	41.8	2.5	34.9	1.6	28.4	1.5	<.0001
≥20 years old	41.5	1.9	33.4	1.4	31.7	2.4	28.8	0.9	28.9	1.3	26.7	1.4	22.3	1.1	<.0001
**Total Sugar intake from Soft Drinks [5]**															
≥2 years old	31.6	1.5	24.2	1.0	22.5	2.1	19.3	0.8	18.1	0.9	18.7	1.1	15.4	0.7	<.0001
2-19 years old	31.7	1.6	24.8	1.8	21.1	1.6	18.2	1.3	16.0	1.0	14.8	1.1	12.5	0.7	<.0001
6-11 years old	27.1	2.7	14.7	1.2	17.6	1.6	12.1	1.0	12.2	1.2	10.9	1.2	10.5	0.9	<.0001
12-19 years old	46.4	2.4	40.9	3.3	31.0	2.9	29.8	2.5	24.3	1.8	22.9	1.9	18.7	1.2	<.0001
≥20 years old	31.6	1.7	24 .0	1.0	23.0	2.3	19.7	0.8	18.8	1.1	19.9	1.2	16.4	0.9	<.0001
**Total Sugar intake from All Beverages**															
≥2 years old	69.8	1.8	60.3	1.8	56.1	2.0	56.3	1.0	55.0	1.4	50.4	1.4	45.8	1.4	<.0001
2-19 years old	84.7	1.8	73.4	2.9	65.8	1.6	66.1	1.3	64.6	1.2	55.9	1.5	47.9	1.2	<.0001
6-11 years old	77.5	3.2	60.5	3.0	62.7	1.9	58.0	1.5	59.0	1.8	52.5	1.4	46.7	1.6	<.0001
12-19 years old	96.2	1.9	88.7	4.5	72.9	2.6	77.6	2.5	73.8	2.2	63.7	2.2	52.8	2.1	<.0001
≥20 years old	64.5	2.2	55.7	1.9	52.8	2.4	52.9	1.0	51.8	1.4	48.6	1.4	45.2	1.6	<.0001
**Total Sugar intake from Total Diet**															
≥2 years old	132.7	1.6	124.1	2.3	119.8	1.9	119.1	1.1	120.0	1.3	111.9	1.3	106.1	1.5	<.0001
2-19 years old	147.8	2.2	138.3	3.0	128.2	1.7	127.0	1.6	128.1	1.8	114.3	1.7	108.3	2.1	<.0001
6-11 years old	147.3	5.3	133.2	3.5	131.3	3.1	123.4	1.7	129.8	2.1	117.5	2.4	111.2	2.5	.0001
12-19 years old	157.2	2.8	152.3	4.9	134.5	2.8	138.1	2.5	135.0	3.4	119.8	2.6	114.2	4.0	<.0001
≥20 years old	127.3	1.7	119.1	2.6	32.6	2.5	116.4	1.3	117.2	1.5	111.2	1.3	105.4	1.6	<.0001

[1] Mean ± standard error (SE) is shown for all values. [2] Data source: NHANES dietary data over 7 cycles (2003-2016) was used (n=57,026; n=5534 children aged 2-5, n=7378 children aged 6-11, n=10,178 children aged 12-18, n=33,936 adults aged ≥20). [3] Estimates for adults aged ≥20 years were age-adjusted by the direct method to the year 2010 US Census Population. [4] All tests for significance for a linear trend were obtained using linear regression for survey data. [5] SSBs (sugar-sweetened beverages) were defined as soft drinks, sports drinks, energy drinks, and fruit drinks, excluding sports beverages with protein, sweetened teas/coffees, and other items. Soft drinks were defined as regular sweetened carbonated soda. US, United States; SE, standard error; SSBs, sugar-sweetened beverages.

Table S3. Percentage SSB Consumers/Non-consumers of Total US Population over Time by Age Groups and Tiers of SSB Consumption [1-3]

	2003-2004		2005-2006		2007-2008		2009-2010		2011-2012		2013-2014		2015-2016		p-value[4]
**Ages ≥2 years (n)**	7880		8112		8448		8943		7852		7972		7819		
	**%**	**SE**	**%**	**SE**	**%**	**SE**	**%**	**SE**	**%**	**SE**	**%**	**SE**	**%**	**SE**	
0 ounces	40.3	1.3	47.8	1.2	48.4	1.3	52.5	1.0	51.2	1.5	55.3	1.2	58.2	1.3	<.0001
>0-12 ounces	12.5	0.5	13.2	0.7	13.7	0.7	12.7	0.5	13.5	0.5	13.1	0.6	14.3	0.8	.4768
>12-24 ounces	21.6	0.7	17.3	0.7	18.1	0.6	17.4	0.6	18.1	0.9	17.3	0.7	15.8	0.6	<.0001
>24 ounces	25.6	1.0	21.7	1.2	19.8	1.4	17.4	0.4	17.2	0.8	14.3	0.8	11.7	0.5	<.0001
**Ages 2-19 years (n)**	3700		3949		3109		3280		3132		3020		2900		
	**%**	**SE**	**%**	**SE**	**%**	**SE**	**%**	**SE**	**%**	**SE**	**%**	**SE**	**%**	**SE**	
0 ounces	23.1	1.7	31.6	1.6	33.8	1.7	39.8	1.4	37.5	2.2	45.8	1.3	45.8	1.2	<.0001
>0-12 ounces	20.9	1.0	21.0	1.4	24.1	1.2	21.3	1.0	23.1	1.2	22.0	0.7	24.5	1.6	.2086
>12-24 ounces	26.9	1.1	22.8	0.9	21.7	1.3	21.3	1.6	22.9	1.3	18.8	0.9	19.5	0.9	<.0001
>24 ounces	29.1	1.6	24.6	1.9	20.4	1.1	17.6	1.3	16.4	1.1	13.3	1.0	10.1	0.9	<.0001
**Ages ≥20 years (n)**	4180		4163		5336		5663		4720		4952		4919		
	**%**	**SE**	**%**	**SE**	**%**	**SE**	**%**	**SE**	**%**	**SE**	**%**	**SE**	**%**	**SE**	
0 ounces	46.5	1.3	53.6	1.3	53.5	1.3	56.9	1.0	55.8	1.5	58.4	1.3	62.2	1.4	<.0001
>0-12 ounces	9.5	0.5	10.5	0.7	10.0	0.7	9.7	0.6	10.3	0.5	10.2	0.6	11.0	0.7	.6809
>12-24 ounces	19.7	0.7	15.3	1.0	16.9	0.9	16.0	0.7	16.5	0.8	16.8	0.8	14.6	0.7	.0002
>24 ounces	24.3	1.0	20.6	1.2	19.6	1.7	17.4	0.6	17.4	0.9	14.7	0.9	12.2	0.7	<.0001

[1] Total N and mean percentage ± standard error (SE) are shown for all values. [2] Data source: NHANES dietary data over 7 cycles (2003-2016) was used n=57,026; 23,090 children aged 2-19, n=33,936 adults aged ≥20). [3] SSBs (sugar-sweetened beverages) were defined as soft drinks, sports drinks, energy drinks, and fruit drinks, excluding sports beverages with protein, sweetened teas/coffees, and other items. [4] Estimates for adults aged ≥20 years were age-adjusted by the direct method to the year 2010 US Census Population. [5] Tests for significance for a linear trend were obtained using logistic regression for survey data. SSBs, sugar-sweetened beverages; US, United States; SE, standard error.
